# A Novel Mechanism of Bacterial Toxin Transfer within Host Blood Cell-Derived Microvesicles

**DOI:** 10.1371/journal.ppat.1004619

**Published:** 2015-02-26

**Authors:** Anne-lie Ståhl, Ida Arvidsson, Karl E. Johansson, Milan Chromek, Johan Rebetz, Sebastian Loos, Ann-Charlotte Kristoffersson, Zivile D. Békássy, Matthias Mörgelin, Diana Karpman

**Affiliations:** 1 Department of Pediatrics, Clinical Sciences Lund, Lund University, Lund, Sweden; 2 Division of Infection Medicine, Clinical Sciences Lund, Lund University, Lund, Sweden; University of Illinois, UNITED STATES

## Abstract

Shiga toxin (Stx) is the main virulence factor of enterohemorrhagic *Escherichia coli*, which are non-invasive strains that can lead to hemolytic uremic syndrome (HUS), associated with renal failure and death. Although bacteremia does not occur, bacterial virulence factors gain access to the circulation and are thereafter presumed to cause target organ damage. Stx was previously shown to circulate bound to blood cells but the mechanism by which it would potentially transfer to target organ cells has not been elucidated. Here we show that blood cell-derived microvesicles, shed during HUS, contain Stx and are found within patient renal cortical cells. The finding was reproduced in mice infected with Stx-producing *Escherichia coli* exhibiting Stx-containing blood cell-derived microvesicles in the circulation that reached the kidney where they were transferred into glomerular and peritubular capillary endothelial cells and further through their basement membranes followed by podocytes and tubular epithelial cells, respectively. In vitro studies demonstrated that blood cell-derived microvesicles containing Stx undergo endocytosis in glomerular endothelial cells leading to cell death secondary to inhibited protein synthesis. This study demonstrates a novel virulence mechanism whereby bacterial toxin is transferred within host blood cell-derived microvesicles in which it may evade the host immune system.

## Author Summary

Shiga toxin-producing enterohemorrhagic *Escherichia coli* are non-invasive bacteria that, after ingestion, cause disease by systemic release of toxins and other virulence factors. These infections cause high morbidity, including hemolytic uremic syndrome with severe anemia, low platelet counts, renal failure, and mortality. The most common clinical isolate is *E. coli* O157:H7. In 2011 an *E. coli* O104:H4 strain caused a large outbreak in Europe with high mortality. After Shiga toxin damages intestinal cells it comes in contact with blood cells and thus gains access to the circulation. In this study we have shown that the toxin is released into circulating host blood cell-derived microvesicles, in which it retains its toxicity but evades the host immune response. Our results suggest that these microvesicles can enter target organ cells in the kidney and transfer toxin into these cells as well as between cells. Such a mechanism of virulence has not been previously described in bacterial infection.

## Introduction

Shiga toxin (Stx) is the major virulence factor of enterohemorrhagic *Escherichia coli* (EHEC). EHEC are non-invasive bacteria [1] causing gastrointestinal infection presenting with diarrhea, hemorrhagic colitis and in severe cases leading to hemolytic uremic syndrome (HUS) characterized by thrombocytopenia, microangiopathic hemolytic anemia and acute renal failure. The renal cortical lesions affect both glomeruli and tubuli. In glomeruli the lesion is termed thrombotic microangiopathy presenting with glomerular capillary endothelial cell damage and formation of microthrombi [2]. In tubuli extensive apoptosis has been described [3]. The tubular damage can be reproduced in mouse models after infection with EHEC [4–6] or intraperitoneal injection of Stx2 and lipopolysaccharide (LPS) [7]. Mice orally infected with EHEC develop systemic and neurological symptoms 7–8 days after inoculation [8] with extensive intestinal and renal pathology, the latter with fibrinogen deposition in glomeruli, as well as marked apoptosis of both tubular and glomerular cells [3,6,8,9]. Laboratory investigation demonstrated fragmented red blood cells, thrombocytopenia and elevated creatinine [5,8]. Thus EHEC-infected mice exhibit clinical and pathological findings that mimic certain aspects of human infection and HUS. Using isogenic strains of *E. coli* O157:H7 these findings were most specifically attributed to the strain’s production of Stx [8].

In order for cells to be affected by Stx, the toxin needs to first bind to its receptor, globotriaosylceramide (Gb3) [10] via its B-binding subunits, followed by endocytosis of the holotoxin. Intracellularly toxin is transported to the endoplasmic reticulum [11] where the A-subunit binds to ribosomes and cleaves an adenine base from 28S rRNA of the 60S ribosomal subunit [12], thus inhibiting protein synthesis. The presence of a glycolipid receptor capable of binding Stx has been considered essential for predicting which cells the toxin will affect [13–16]. However, human intestinal cells may be damaged by Stx even in the absence of the toxin receptor [17] and murine glomeruli, lacking the Gb3 receptor, develop toxin-related injury in vivo [18–20]. These findings suggest that Stx may also mediate cytotoxicity to target organ cells in a Gb3 receptor-independent manner.

The means by which Stx affects target organ cells has not been clarified. Negligible amounts of free toxin are present in the circulation during HUS [21]. The toxin circulates preferentially in cell-bound form, mainly bound to platelets, neutrophils and monocytes [22,23]. In order to affect renal cells the toxin would first have to be released from blood cells possibly due to higher affinity for renal endothelial cells [24,25]. A prerequisite for this to occur would be that the toxin remains on the cell membrane and does not undergo receptor-mediated endocytosis. Evidence has, however, shown that the toxin does undergo endocytosis in platelets [26]. Furthermore, stimulation of blood cells with Stx leads to the release of platelet and leukocyte-derived microvesicles [22,27] with surface-bound tissue factor [22] as well as C3 and C9 deposition [27], contributing to a pro-thrombotic state.

Microvesicles are small (<1 μm), pro-inflammatory vesicles shed by host cells during activation and apoptosis. They contain surface markers of their parent cells [28,29]. Microvesicles mediate cell-to-cell communication by transferring cell surface receptors [30,31], chemokines [32], mRNAs [33] and microRNAs [34] from the cell of origin to target cells. They circulate in elevated levels during EHEC-associated HUS [22,27,35].

In this study we investigated the possibility that blood cell-derived microvesicles contain Stx that is thus transferred into target organ cells and if Stx within microvesicles retains cytotoxic potential. We found Stx within blood cell-derived microvesicles in the circulation of patients with HUS and of mice infected with *E. coli* O157:H7, and within human and murine renal tissue. In vitro studies showed that human blood cell-derived microvesicles containing Stx underwent endocytosis in human glomerular endothelial cells where microvesicles released the toxin and lead to cell death by inhibition of protein synthesis. Bacterial toxin can thus be transferred within host cell-derived microvesicles and evade the host response.

## Results

### Patients with HUS have circulating microvesicles containing Stx2

High levels of platelet and leukocyte-derived microvesicles were detected in plasma from patients with HUS (n = 13, Patients 1–13 in S1 Table, supporting information) by flow cytometry (Table 1). Most of the microvesicles were of platelet origin. Similarly, red blood cell (RBC)-derived microvesicles were detected in plasma from patients with HUS (n = 6, Patients 6–11).

**Table 1:** Numbers and cellular origin of microvesicles containing Stx2 in plasma from patients and controls.

	Platelet-derived microvesicles (x10^3^/mL)		Monocyte-derived microvesicles (x10^3^/mL)		Neutrophil-derived microvesicles (x10^3^/mL)		RBC-derived microvesicles (x10^3^/mL)	
		Stx2-positive		Stx2-positive		Stx2 positive		Stx2 positive
**HUS Acute phase (n = 13)**	1697 (315–3900)***	453 (57–855)***	603 (99–1509)**	187 (30–354)**	517 (45–1794)***	135 (38–222)***	543 (240–1182)^a^	51 (12–120)*^a^
**Recovery (n = 12)**	154 (63–241)	0	43 (6–212)	0	13 (3–91)	0	114 (90–618)^b^	0^b^
**HC (n = 5)**	215 (108–574)	0 (0–212)^c^	102 (35–489)	0 (0–99)^c^	94 (25–324)	0 (0–72)^c^	NA	NA
**Controls (n = 10)** ^**d**^	123 (80–171)	0	42 (21–56)	0	28 (15–38)	0	5 (0–8)	0
**Renal failure controls (n = 2)**	130–282	0	31–96	0	58–133	0	NA	NA

Samples from pediatric HUS patients (1–12) were available during the acute phase of disease and after recovery whereas samples from patient 13 and patients with hemorrhagic colitis (15–19) were only available during the acute phase of disease. Data are expressed as median and (range) of circulating microvesicles positive for each membrane specific marker (CD42b for platelets, CD38 for monocytes and CD66 for neutrophils). *** Denotes *P* value <0.001, ** *P*<0.01 and *P<0.05 when comparing microvesicles in plasma from HUS patients with recovery.

^a^, HUS patients analyzed for CD235a-positive red blood cells (RBC)-derived microvesicles (n = 6, not all patients were analyzed). Significantly higher levels were detected during the acute phase compared to controls (n = 6), *P*<0.001).

^b^, Recovery samples analyzed for RBC-derived microvesicles (n = 3).

^c^, Two of the patients with HC had detectable levels of microvesicles containing Stx2.

^d^, Pediatric controls (n = 4) analyzed for platelet (CD42b), monocyte (CD38) and neutrophil (CD66)-derived microvesicles. Adult controls (n = 6) analyzed for RBC/CD235a-derived microvesicles. NA: not analyzed.

Significantly higher levels of circulating microvesicles (derived from platelets and leukocytes), and microvesicles containing Stx2 (derived from platelets, leukocytes and RBCs), were detected in plasma during the acute phase of HUS compared to after recovery (Table 1). Levels of platelet and leukocyte microvesicles at recovery were similar to those found in controls.

Microvesicles levels were slightly elevated in patients with hemorrhagic colitis (n = 5, Patients 15–19) and two of these patients exhibited Stx2 in microvesicles from platelets and leukocytes. No Stx2 was detected in microvesicles from the controls (n = 10) or patients with acute renal failure (n = 2), as expected. In the absence of membrane permeabilization with saponin no Stx2 was detected on the surface of microvesicles.

### Blood-cell derived microvesicles were demonstrated in the kidney of a patient with HUS

**Fig 1:**
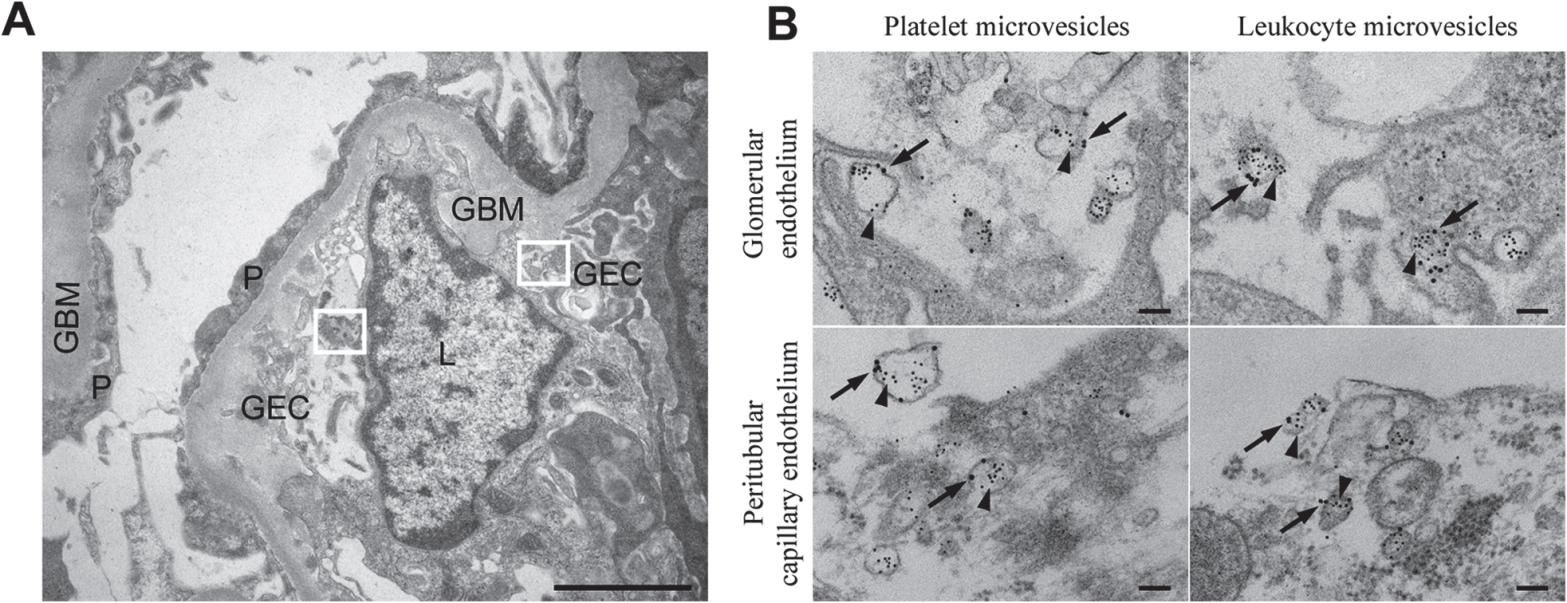
Stx2-containing blood cell-derived microvesicles detected in the renal cortex of a HUS patient. (**A**) Ultramorphology showing an overview from the renal cortex of a patient with HUS (patient 14) including a glomerular capillary with two boxes magnified in (B). Scale bar: 2 μm. P: podocyte, GEC: glomerular endothelial cell, GBM: glomerular basement membrane, L: leukocyte. (**B**) Renal cortex of a the same HUS patient labeled with anti-Stx2 (5 nm, arrowhead), anti-CD42b (10 nm, arrow) to detect platelet-derived microvesicles or anti-CD45 (10 nm, arrow) to detect leukocyte-derived microvesicles. Microvesicles were defined by their size (shed microvesicles: ≤ 1μm, apoptotic bodies: 1–5 μm) and their cellular origin based on the presence of platelet- or leukocyte-markers. Stx2-containing microvesicles were detected in glomerular- and peritubular capillary endothelium. The two upper panels are magnifications of the two boxes depicted in (A). Scale bar: 100 nm.

A renal cortical biopsy from a patient with *E. coli* O157:H7-induced HUS (Patient 14) was examined by immune-electron microscopy labeled for Stx2. Thirty cellular profiles were examined in glomerular and tubular regions. Numerous platelet- and leukocyte-derived microvesicles labeled for Stx2 were demonstrated adjacent to and within endothelial cells (Fig. 1A,B). Altogether, 0–10 platelet- or leukocyte-derived microvesicles containing Stx2 were demonstrated per cellular profile. No specific binding of control antibodies was observed in the tissue in general, and specifically on or within microvesicles.

### Stx2 is present in circulating microvesicles from EHEC-infected and Stx2-treated mice

**Fig 2:**
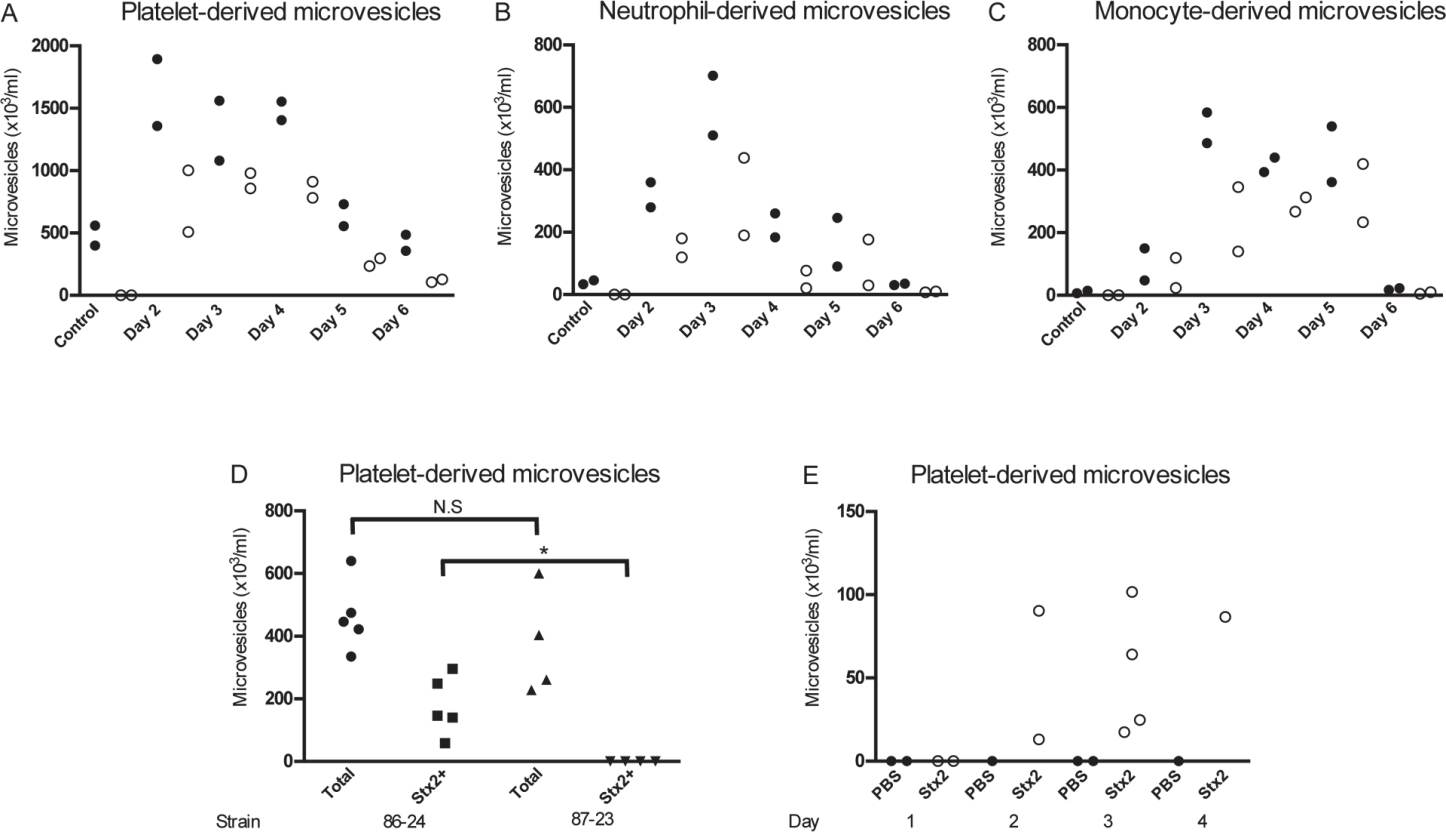
Stx2-containing blood cell-derived microvesicles were detected in the circulation of mice infected with *E. coli* O157:H7. Microvesicles were isolated from whole blood of BALB/c mice (n = 10) inoculated with the Stx2-producing *E. coli* O157:H7 strain and analyzed by flow cytometry. Samples were taken between days 2 and 6 after infection. Microvesicles were labeled with anti-Stx2 and anti-mouse CD41:APC (**A**) to identify platelet-derived microvesicles, anti-mouse Ly-6G:PE (**B**) to identify neutrophil-derived microvesicles or a combination of anti-mouse CD45R/B222:PerCp-Cy5.5 and anti-mouse Ly-6G:PE (**C**) to identify monocyte-derived microvesicles. All microvesicles were labeled with cell-specific and Stx2 antibodies. Stx2-containing microvesicles were mostly detected on days 2–5 after infection. No labeling of the Stx2 antibody was detected in the non-infected mice (n = 2, day 6). (**D**) Platelet-derived microvesicles (labeled with anti-mouse CD41:APC) and stained for Stx2 were isolated from mice inoculated with Stx2-producing *E. coli* O157:H7 strain 86–24 and the isogenic non-Stx producing *E. coli* O157:H7 strain 87–23 on day 3 post-inoculation. Only mice inoculated with strain 86–24 exhibited Stx within microvesicles. * Denotes *P*<0.05. (**E**) Platelet-derived microvesicles labelled for Stx2 were isolated from mice injected with Stx2 i.p. or PBS controls. An increase in microvesicles was noted in Stx-treated mice 3–4 days after treatment.

The findings in HUS patients were further studied in EHEC-inoculated mice. BALB/c mice (n = 10) were infected with the Stx2-producing *E. coli* O157:H7 strain 86–24. Blood was drawn from two mice each day between days 2–6 after inoculation, before any symptoms developed, and plasma levels of circulating microvesicles were measured by flow cytometry and compared to control mice (n = 2, samples taken on day 6). Plasma taken between days 2–5 showed considerably higher levels of circulating platelet- and leukocyte-derived microvesicles (Fig. 2) compared to the controls. Stx2 was detected in microvesicles released from platelets (Fig. 2A), neutrophils (Fig. 2B) and monocytes (Fig. 2C) at all time points (not assayed for RBCs). No Stx2 was detected in microvesicles from control mice.

In a separate experiment mice were inoculated with the Stx2-producing *E. coli* O157:H7 strain 86–24 (n = 5) and the isogenic non-Stx producing *E. coli* O157:H7 strain 87–23 (n = 4) and sacrificed 3 days after inoculation. There was no statistical difference between the strains regarding the total number of platelet-derived microvesicles but only microvesicles from mice infected with the Stx2-producing strain contained Stx, as expected (Fig. 2D). Similarly, mice injected intraperitoneally with Stx2 (n = 9) also exhibited an increase in Stx2 within platelet-derived microvesicles on days 2–4 post-injection (Fig. 2E), albeit at lower concentrations than in EHEC-infected mice.

### In vivo transfer of Stx2 to kidney cells within blood cell-derived microvesicles

**Fig 3:**
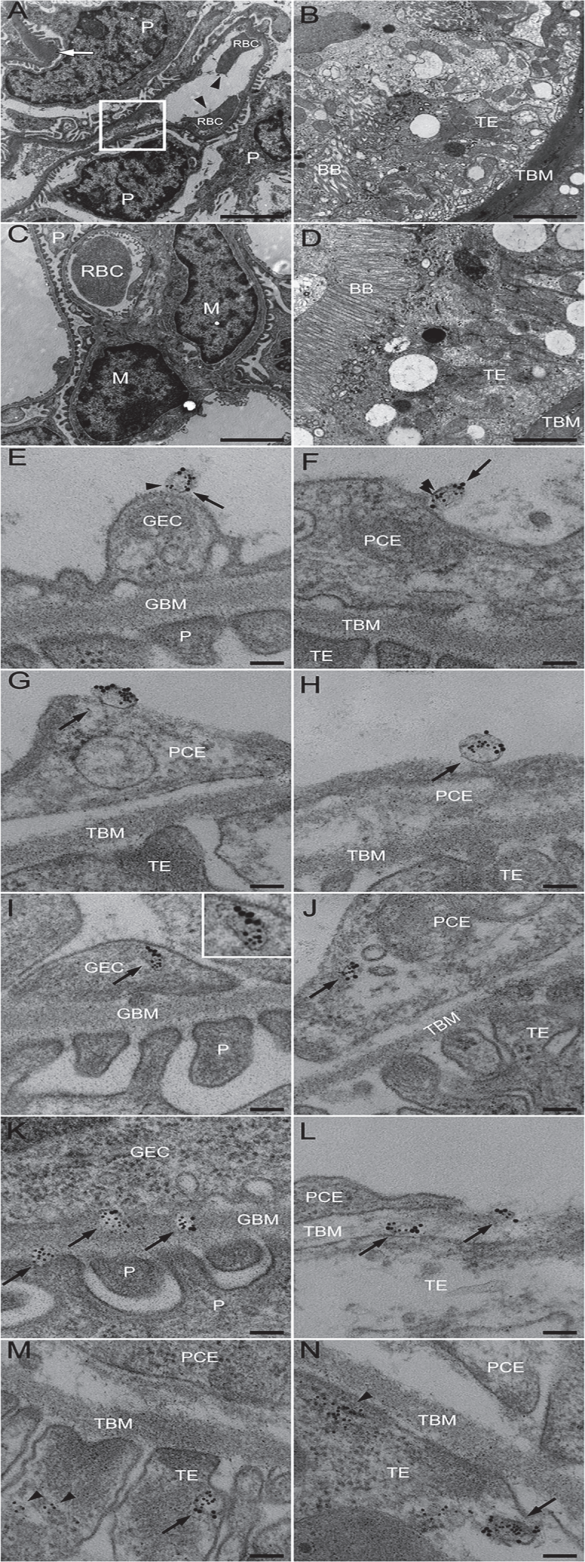
Ultramorphology of the renal cortex in mice infected with *E. coli* O157:H7. Representative overviews of glomerular (**A**, box depicts an area of endothelial damage with detachment from the basement membrane, arrow indicates thickening of the glomerular basement membrane and arrowheads point to fragmented RBCs in a glomerular capillary) and tubular (**B**) damage in infected mice. These are compared to a glomerulus (**C**, showing thin basement membranes and normal round RBC in a glomerular capillary) and tubulus (**D**) from uninfected mice, all taken on day 6 post-inoculation. Samples were co-incubated with rabbit anti-Stx2 (5 nm, arrowhead) and rat anti-mouse CD41 (10 nm, detects platelet-derived microvesicles, arrow) or rat anti-mouse CD45 (10 nm, detects leukocyte-derived microvesicles, arrow) showing binding of Stx2-containing microvesicles to glomerular (**E**, showing platelet microvesicle, **F**, showing leukocyte microvesicle) and peritubular capillary endothelial cells (**G**, platelet microvesicle, **H**, leukocyte microvesicle). Stx2-containing microvesicles were detected within glomerular (**I**, platelet microvesicle, see inset for enlargement) and peritubular (**J**, leukocyte microvesicle) capillary endothelial cells, glomerular (**K**) and tubular (**L**) basement membranes (both showing platelet microvesicles), within podocytes (**K**) and tubular epithelial cells (**M**, leukocyte microvesicle, **N**, platelet microvesicle). E-N taken on day 4 post-inoculation. Scale bar 100 nm. For detailed enlargement of microvesicles in panels I-N see S2 Fig. (in supporting information). P: podocyte, RBC: red blood cell, TE: tubular epithelial cell, BB: brush border, TBM: tubular basement membrane, M: mesangial cell, GEC: glomerular endothelial cell, GBM: glomerular basement membrane, PCE: peritubular capillary endothelium.

Electron microscopy of kidneys from mice infected with *E. coli* O157:H7 showed extensive glomerular endothelial (Fig. 3A) and tubular epithelial (Fig. 3B) damage on Day 6 post-inoculation, in comparison to controls (Fig. 3C,D). Kidneys from infected and control mice were examined for the presence of Stx2-containing platelet- and leukocyte-derived microvesicles (≤1 μm) on days 2–6 after inoculation. On days 3–6 post-inoculation Stx2-containing platelet- and leukocyte-derived microvesicles were observed on (Fig. 3E-H) and within (Fig. 3I) glomerular endothelial cells as well as within endothelial cells in peritubular capillaries (Fig. 3J). Furthermore, Stx2 containing blood cell-derived microvesicles were identified within the glomerular (Fig. 3K) and tubular basement membranes (Fig. 3L) and within podocytes (Fig. 3K) and tubular epithelial cells (Fig. 3M,N). At all localizations Stx2 was identified within microvesicles as well as in free form (Fig. 3N). Quantification of Stx2 containing platelet- and leukocyte-derived microvesicles was carried out in 50 cell profiles in the glomerular and peritubular capillary endothelium as well as in the tubular epithelium in infected and control mice as presented in Table 2. The results indicate that most Stx2-containing microvesicles were of platelet origin and localized to the glomerular and peritubular capillary endothelium in the infected mice. In the non-infected mice minimal background signal was observed (0–3 gold particles). Control antibodies bound minimally and unspecifically.

**Table 2:** Numbers and cellular origin of Stx-positive microvesicles in mice infected with *E. coli* O157:H7.

	Glomerular endothelium		Peritubular capillary endothelium		Tubular epithelium	
Day	Stx2 + Platelet microvesicles	Stx2 + Leukocyte microvesicles	Stx2 + Platelet microvesicles	Stx2 + Leukocyte microvesicles	Stx2 + Platelet microvesicles	Stx2 + Leukocyte microvesicles
2	33 (18–44)	21 (10–25)	21 (18–24)	13 (11–15)	9 (7–14)	7 (5–10)
3	49 (29–60)	23 (15–32)	43 (39–52)	20 (17–22)	11 (6–14)	8 (6–14)
4	53 (41–66)	22 (14–30)	46 (40–56)	22 (16–28)	13 (10–19)	10 (7–18)
5	57 (38–75)	24 (14–31)	48 (38–55)	22 (16–29)	13 (8–25)	9 (7–19)
6	55 (34–65)	25 (19–31)	47 (39–62)	24 (19–31)	12 (9–20)	10 (8–20)
6 (non-infected)^a^	1 (1–2)	2 (1–3)	1 (0–2)	1 (1–2)	0	0

Data are expressed as median and (range) of microvesicles positive for each specific surface marker and Stx2. Values are derived from 50 cellular profiles from two independent experiments.

^a^, These uninfected mice were sacrificed on Day 6, the infected mice were sacrificed on Days 2–6.

### Stx2 induced release of blood cell-derived microvesicles containing Stx2 in vitro

**Fig 4:**
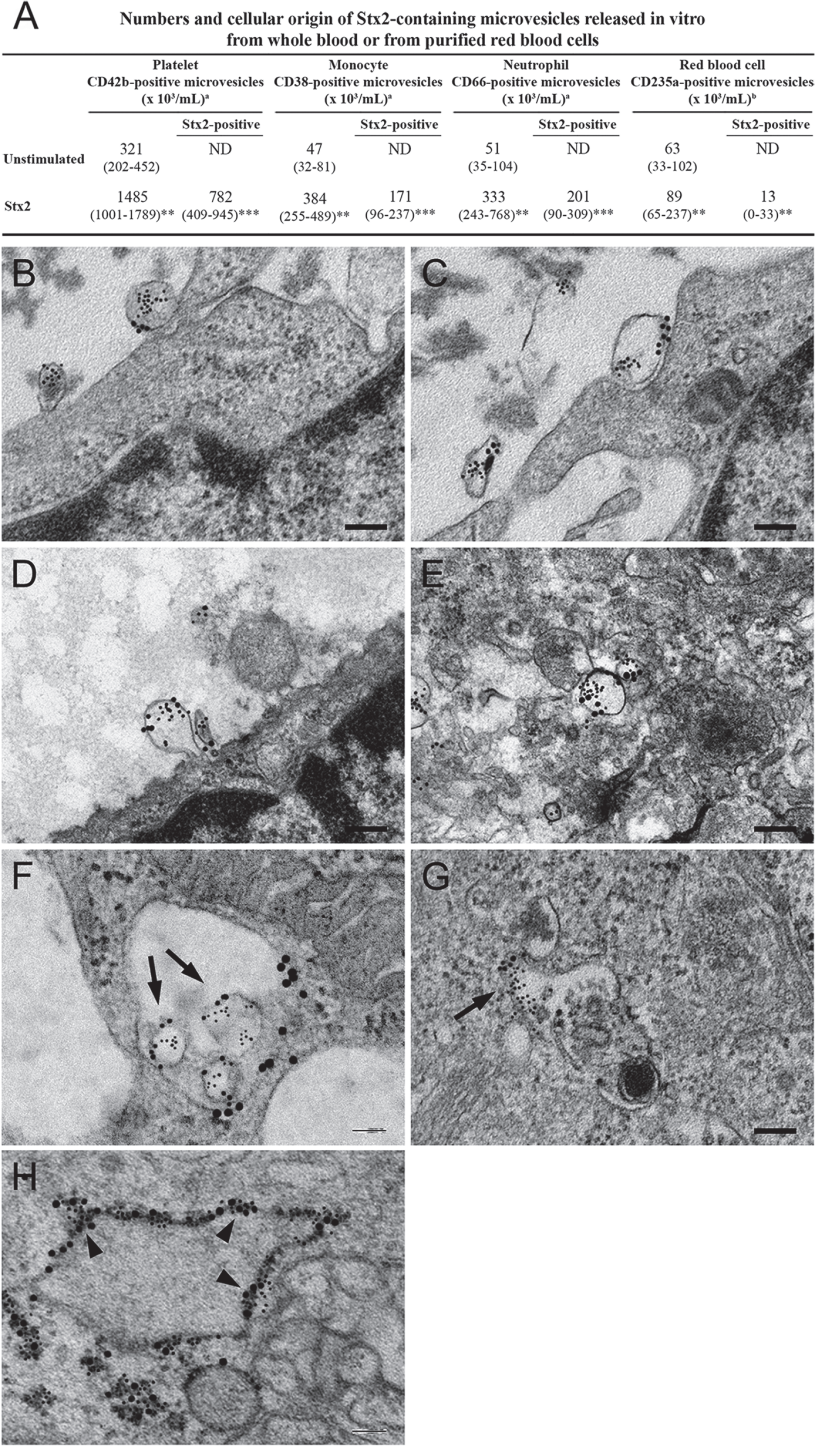
Blood cell-derived microvesicles transfer Stx2 to glomerular endothelial cells in vitro. (**A**) Data are expressed as median and (range) of microvesicles positive for each membrane specific marker/mL of plasma. ^a^, Microvesicles released from whole blood (n = 6). ^b^, Microvesicles released from purified RBCs (n = 10). *** Denotes *P* value <0.001, ** *P*<0.01 and * *P*<0.05 when comparing microvesicle generation in whole blood stimulated with Stx2 with unstimulated whole blood, or RBCs stimulated with Stx2 with unstimulated RBCs. ND: not detected. (**B—H**) Conditionally immortalized glomerular endothelial cells (CiGEnC) were incubated with microvesicles isolated from Stx2-stimulated whole blood for 1–24 h. Cells were stained with rabbit anti-Stx2 (5 nm gold particles) and mouse anti-human CD42b or mouse anti-human CD45 to identify platelet- or leukocyte-derived microvesicles, respectively (10 nm gold particles). After 1 h of incubation Stx2-containing platelet- (**B**, **C**) and leukocyte- (**D**) derived microvesicles bound to and appeared to fuse with the cell membrane or were found in the cell cytoplasm (**E**, platelet microvesicle). At 3 h Stx2-containing microvesicles are demonstrated within early endosomes, see arrows (**F**, leukocyte microvesicle, anti-early endosome antibody detected with 20 nm gold particles). Endosome membranes were disrupted after 12 h, see arrow (**G**, leukocyte microvesicle) and Stx2 was bound to ribosomes (arrowheads) in the cytoplasm after 24 h (**H**, anti-ribosome antibody detected with 20 nm gold particles).

Microvesicles containing Stx2 were detected in whole blood stimulated with Stx2 by flow cytometry. Stx2 induced a significant increase in the release of microvesicles compared with the phosphate-buffered saline (PBS)-treated samples (Fig. 4A). Stx2 was detected in microvesicles released from platelets, monocytes and neutrophils. Most microvesicles were of platelet origin. Similarly, purified RBCs stimulated with Stx2 released microvesicles in which Stx2 was detected. No Stx2 was detected within microvesicles from the PBS-treated samples, or on the surface of microvesicles.

### Blood cell-derived microvesicles transferred Stx2 to glomerular endothelial cells in vitro

Transfer of Stx2 to glomerular endothelial cells by blood cell-derived microvesicles was investigated by incubation of conditionally immortalized glomerular endothelial cells (CiGEnC) with Stx2-containing microvesicles and visualization by electron microscopy (n = 2). Results showed that platelet- and leukocyte-derived Stx2-containing microvesicles bound to (Fig. 4B,C) and fused with CiGEnC after 1h (Fig. 4D) and were demonstrated within the cell cytoplasm (Fig. 4E) or in early endosomes (Fig. 4F) after 3h. At 12h the membranes of the early endosomes were disrupted and free Stx2 was visualized in the cytoplasm (Fig. 4G). After 24h Stx2 was bound to ribosomes in the cytoplasm (Fig. 4H). No specific binding of the control antibodies was detected.

### Stx2 transferred within blood cell-derived microvesicles affected cell viability

**Fig 5:**
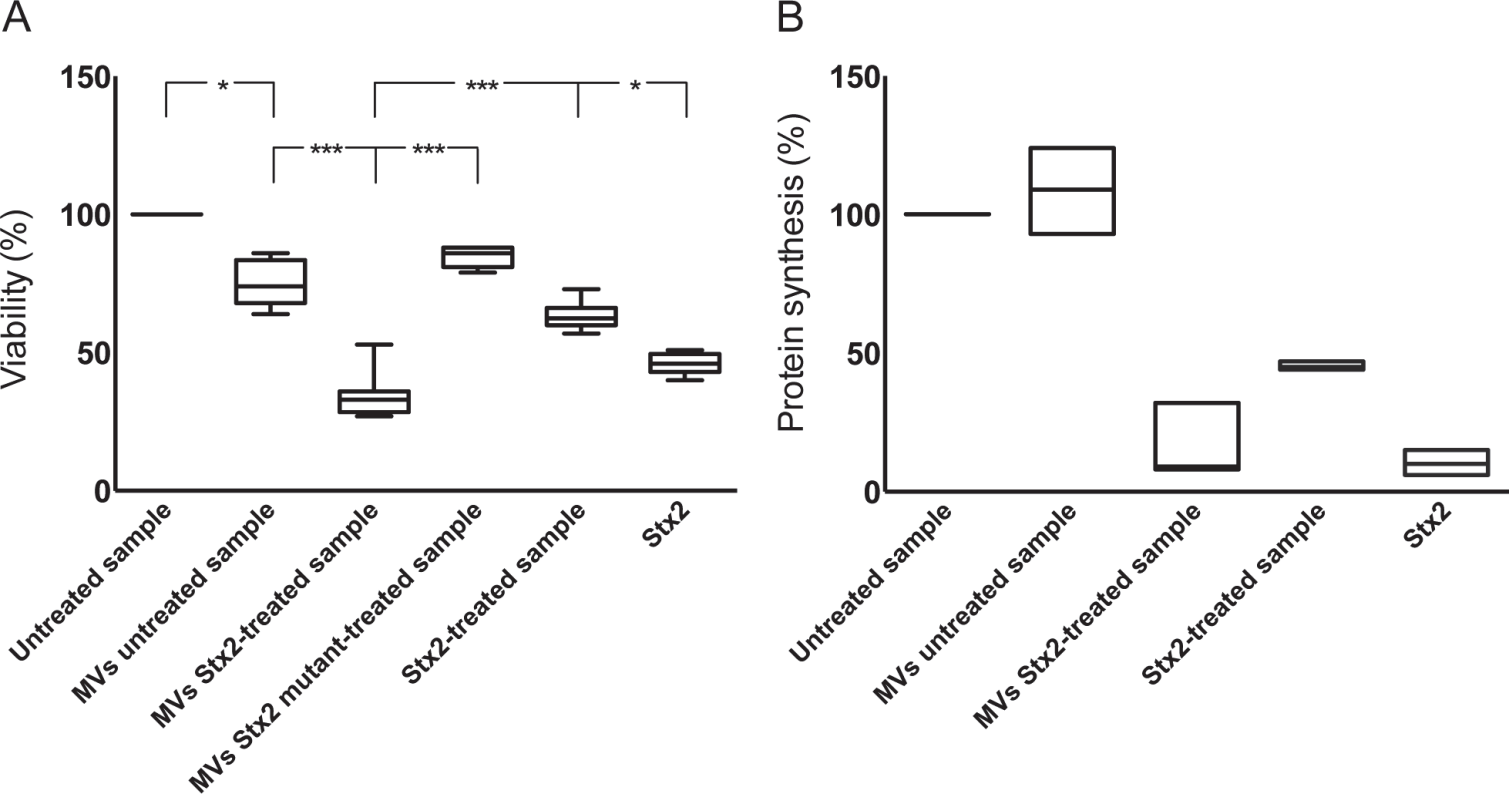
Microvesicles containing Stx2 affected the viability and inhibited protein synthesis in CiGEnC. The effect of Stx2-containing microvesicles on cell viability was examined by use of a crystal violet assay (**A**). Data are expressed as percentage of control cell viability defined as 100%. Representative data from five experiments are depicted as the median of triplicate wells. *** Denotes *P* value <0.001 and * *P*<0.05. (**B**) Protein synthesis was measured as the incorporation of [^35^S]-methionine into total protein. Data are expressed as percent and 100% represents the untreated cells incubated under the same conditions but without microvesicles. All experiments were done in duplicate and the experiment was repeated three times. MVs untreated sample: whole blood was exposed to PBS for 1 hr, microvesicles (MVs) were isolated and incubated with CiGEnC for 36 hr; MVs Stx2-treated sample: isolated from whole blood treated with Stx2 (200 ng/mL diluted in PBS) for 36h; MVs Stx2 mutant-treated sample: isolated from whole blood treated with Stx2 mutated in the catalytic A subunit for 36h: Stx2-treated sample: purified Stx2 was exposed to washing steps similar to microvesicles before exposure to the cells; Stx2: cells were exposed to pure Stx2 (200 ng/mL) without washing steps.

The cytotoxic effect of Stx2-containing microvesicles was examined by incubation of CiGEnC with microvesicles isolated from Stx2-treated whole blood. These microvesicles induced significantly more cell death compared to microvesicles from untreated blood (*P*<0.001, Fig. 5A). After 36h incubation the number of viable cells was reduced to a median of 30% (range 24–53%, n = 5) in the samples incubated with microvesicles from the Stx2-treated blood samples whereas a median of 75% (range 63–87%) of the cells treated with microvesicles from the unstimulated blood samples were viable in comparison to the untreated cells (defined as 100% viability). Cells treated with microvesicles containing the enzymatically inactive Stx2 mutant showed a slight reduction in viability (median 85%, range 80–88%). CiGEnC incubated with Stx2-treated washed samples (purified Stx2 exposed to washing steps similar to microvesicles before exposure to the cells, but without microvesicles) exhibited 62% (range 57–73%) viability while treatment of the cells with pure Stx2 (without washing steps) reduced viability to 46% (range 40–51%) (Fig. 5A).

### Stx2 transferred within blood cell-derived microvesicles inhibited protein synthesis

To determine if the cytotoxic effect of microvesicles containing Stx2 was associated with inhibited protein synthesis cultured CiGEnC were incubated with Stx2-containing microvesicles and [^35^S]-methionine incorporation was measured. Protein synthesis was reduced to 9% (median, range 8–32%, n = 3, Fig. 5B) in CiGEnC treated with Stx2-containing microvesicles compared to untreated cells. Incubation of cells with Stx2-containing media exposed to washing steps similar to the microvesicles reduced protein synthesis to 44–47% (n = 3, median 45%), respectively, and exposure of cells to purified Stx2 inhibited protein synthesis to 6–15%, (n = 3, median 10%). Cells treated with microvesicles from unstimulated samples showed a slightly increased protein synthesis (median 110%, range 93–124%, n = 3).

## Discussion

In this study we showed that Stx2, after binding to blood cells, was released from these cells within microvesicles and that these microvesicles could thereafter bind to renal glomerular and peritubular capillary endothelial cells and undergo endocytosis. Inside renal cells Stx was released from microvesicles and exerted a cytotoxic effect equivalent to purified toxin. Not all toxin was released from microvesicles, as certain Stx2-containing microvesicles were transferred from cell to cell, even via the glomerular or tubular basement membranes, thus reaching podocytes and tubular epithelial cells. This mechanism of Stx endocytosis would be independent of the toxin Gb3 receptor. The transfer of virulence factors within host blood cell-derived microvesicles is a novel mechanism of bacterial pathogenesis.

Several mechanisms of microvesicle-mediated communication with cells have been described. Microvesicles can bind to cells by expressing specific receptors, by fusion with the cell membrane, by endocytosis and by release of mediators that bind to the cells [36]. Ultramorphological results presented here indicate that Stx2-containing microvesicles underwent endocytosis as they were labeled with blood cell markers within glomerular and peritubular capillary endothelial cells. In vitro experiments using glomerular endothelial cells indicated that microvesicles were either taken up by early endosomes or underwent fusion with the cell membrane. Blood cell-derived microvesicles taken up in endosomes were not destroyed within endothelial cells and could thus transit intact between cells and through basement membranes until the contents of the microvesicle were released intracellularly.

The necessity of the Gb3 or another glycolipid receptor for Stx internalization and the induction of cellular injury has been described [37], and in mice Gb3-deficiency conferred resistance to the effects of Stx administered intravenously [38]. However, it is, as yet, unclear whether Stx binds to and damages human intestinal epithelial cells or gains access to the systemic circulation by other pathways. The toxin is capable of damaging human intestinal cells that lack the receptor [39] and Stx was found within non-Gb3 expressing intestinal cells of a patient infected with EHEC [40]. Furthermore, Stx B subunit was shown to undergo endocytosis by macropinocytosis in a clathrin-independent manner [40]. Another Gb3-independent mechanism of toxin binding was exhibited on neutrophils (lacking the Gb3 and Gb4 receptor) which bound Stx via its A enzymatic subunit [41,42]. Here we demonstrated an alternative Gb3-independent process of toxin uptake mediated by the endocytosis of toxin-containing microvesicles derived from blood cells. The latter process requires the presence of Gb3 in order for the initial binding of Shiga toxin to platelets [43], monocytes [44] and red blood cells [45] to occur.

After inducing hemorrhagic colitis [46] Stx will come in contact with blood cells. The toxin will thus bind to blood cells, including platelets [26,47], leukocytes [23,44,48–50] and possibly RBCs, leading to blood cell activation and membrane blebbing [22]. Shiga toxin may be internalized within platelets [26], macrophages [51] and possibly within other blood cells. The internalized toxin could thus be released within microvesicles. An alternative speculative explanation for the presence of Shiga toxin within microvesicles is the loss of lipid asymmetry in the membrane lipid bilayer typical for microvesicles by which substances bound to lipid receptors on the outer Gb3 or an alternative toxin receptor of a microvesicle may potentially flip to the inside while remaining membrane-bound [52]. Free toxin is minimal in the bloodstream of infected patients [21,53,54] and thus most of the toxin is cell-bound or internalized. We propose, based on the results presented here, that the release of toxin-containing microvesicles from these blood cells will enable the transfer of toxin to its target cells. This seems more likely than the transfer of toxin from one cell to another after binding to its receptor and endocytosis. Toxin within microvesicles will, in this manner, evade host immune recognition and then be taken up by renal cells and induce cell damage.

An interesting finding was that Stx-containing microvesicles were present in both glomerular and peritubular capillary endothelial cells simultaneously. This would indicate that circulating microvesicles affect both cell types thus explaining why intense damage to both glomeruli and tubuli occurs during Stx-mediated HUS [3,55]. This degree of damage to both cell types was also noted in the murine model of infection where glomerular endothelial cell damage would be achieved in a Gb3-independent manner as murine glomerular endothelial cells do not express Gb3 [7]. Similar glomerular findings were noted in murine models of Stx injection [18–20], which could also be explained by an indirect effect of toxin on tubular epithelial cells (which express Gb3 [7]) extending to ischemic damage of the entire nephron. However, our results show that toxin within microvesicles is internalized in non-Gb3 expressing cells and we therefore suggest that the toxin affects cells that do not express the specific glycolipid receptor by this mechanism.

Toxin-containing microvesicles did not appear to be targeted for lysosomal degradation and could thus proceed to affect cell viability after release of contents. Once within a cell free StxA1 will be translocated in a retrograde fashion to the large ribosomal subunit where it inhibits protein synthesis and ultimately leads to cell death [12]. A certain amount of toxin was demonstrated in free form within the cell cytoplasm, enabling cellular damage to occur, while other toxin-containing microvesicles passed through cells without total release of contents. It is thus unclear if microvesicles undergo partial release of their contents while passing within a cell or if total release of microvesicle contents occurs and which mechanisms regulate the process of microvesicle uptake, release of contents and/or transfer to a neighboring cell.

An interesting finding was that mice injected with Stx2 also exhibited the presence of platelet-derived microvesicles containing Stx, although at lower concentrations than mice inoculated with EHEC. EHEC infection will have a more profound effect in mice, as persistent colonization will allow continuous release of toxin, as well as other bacterial virulence factors, followed by a more severe host response [5,8,9]. This would explain why EHEC infection promotes the release of higher numbers of toxin-containing microvesicles. Nevertheless, the murine model using purified toxin and in vitro experiments demonstrate that toxin may bind to blood cells and be released within microvesicles even in the absence of other bacterial or host systemic as well as intestinal factors involved in EHEC infection.

Microvesicles have been well-characterized with regard to their ability to induce thrombosis by expressing phosphatidylserine capable of activating coagulation factors [56] and by expression of tissue factor [22,57,58]. In Stx-mediated HUS they also possess complement deposits indicating that complement activation occurred on the parent cells and reflecting the inflammatory and thrombogenic process occurring during HUS [27]. Here we defined a new mechanism of virulence in this non-invasive bacterial infection showing that microvesicles transfer bacterial toxin to target cells. Thus blood cell derived-microvesicles may play an important role in the development of HUS. Future studies will address the therapeutic option of interference with microvesicle release during EHEC infection. In addition to the damaging effects of microvesicles presented here, microvesicle release may also be a beneficial mechanism by which cells rid themselves of unwanted substances (foreign as well as host-derived). The effects of blocking microvesicle release, particularly from blood cells, will require further study.

## Materials and Methods

### Subjects

Blood samples were available from 9 boys and 7 girls, aged 1–10 years (median 4 years, patients 1–12 and 15–18 in S1 Table, supporting information), treated for EHEC infection at the Department of Pediatrics, Skåne University Hospital, Lund and Malmö. Samples were obtained within three days of admission while all children still had diarrhea and all but four had HUS. Blood samples were also available from two adults with EHEC infection, one with and one without HUS, treated at the Department of Infectious Diseases, Skåne University Hospital (Patients 13 and 19). HUS was defined as hemolytic anemia (hemoglobin levels <100 g/L), thrombocytopenia (platelet counts <140 x 10^9^/L) and acute renal failure. Patients 7–12 were previously described [27,59].

Samples were also available from four pediatric controls, three girls and one boy aged 6–15 years (median 9 years), seen at the outpatient clinic for unrelated conditions and from two patients with acute renal failure. These patients were treated for acute myeloid leukemia (a 13-year-old boy) or for acute renal failure associated with sepsis (an adult male at the Department of Nephrology). Blood was, in addition, obtained from 15 healthy adult volunteers (9 women, 6 men) not using any medications.

A renal cortical biopsy was available from a 13-year-old boy with EHEC-associated HUS (Patient 14). The biopsy was previously described [59] and performed 2 days after the development of HUS. Tissue was prepared for paraffin-embedding according to hospital routines and used in electron microscopy.

### Blood collection and isolation of platelet-free-plasma

Blood was drawn by venipuncture into vacutainer tubes (Becton Dickinson, Franklin Lakes, NJ) containing 0.5 mL of 0.129 M sodium citrate (Becton Dickinson) through an intravenous cannula or a butterfly needle (Terumo Medical products Hangzhou CO, Hangzhou, China) with low tourniquet. The first tube following venipuncture was discarded. Within 60 minutes of blood collection, blood cells were removed by centrifugation (2600×g, 15 min, 20°C) followed by a second centrifugation step (9900×g, 5 min, 20°C) to obtain platelet-free-plasma (PFP). PFP was carefully removed without disturbing the buffy coat, divided in 200 μL aliquots and stored at—80°C until analyzed for the presence of microvesicles.

### Mice

BALB/c mice were bred in the animal facilities of the Department of Microbiology, Immunology and Glycobiology, Lund University. Both female and male mice were used at 9–14 weeks of age. Studies from our group and others have shown that BALB/c mice develop symptoms after EHEC infection [60,61] and Stx injection [62].

### Bacteria

The Stx2-producing *E. coli* O157:H7 strain 86–24 and the isogenic non-Stx producing *E. coli* O157:H7 strain 87–23 (kindly provided by A. D. O'Brien, Uniformed Services University of the Health Sciences, Bethseda, MD) were previously characterized [9]. Streptomycin-resistant derivatives of these strains were isolated as previously described [8]. Bacteria were grown overnight at 37°C in Luria-Bertani broth supplemented with 50 μg/mL Streptomycin sulfate (Sigma-Aldrich, St. Louis, MO), harvested by centrifugation, washed in sterile phosphate buffered saline (PBS, pH 7.4, Medicago AB, Uppsala, Sweden) and re-suspended in 20% (w/v) sucrose and 10% (w/v) NaHCO_3_ in sterile water at a concentration of 10^9^ colony forming units (CFU)/mL. The bacterial concentration was confirmed by plating serial dilutions of the bacterial suspension on Luria-Bertani agar plates. Each mouse was infected orally with 10^8^ CFU in 100 μl solution.

### Infection protocol

The *E. coli* O157:H7 infection protocol has been previously described [6]. Fecal samples were collected on day one, three and five after inoculation to confirm colonization. In this infection model symptoms usually develop on day 7–8 after inoculation. Mice were anesthetized with isoflurane (Forene, Abbott, Wiesbaden, Germany), blood was collected from vena saphena and by heart puncture, and the mice were then sacrificed by cervical dislocation at day 2, 3, 4, 5 or 6 after inoculation. For analysis of microvesicles and blood urea nitrogen (BUN, QuantiChrom urea assay kit, Bioassay Systems, Hayward, CA), blood was collected into citrated syringes and treated with sterile-filtered paraformaldehyde (PFA, Histolab, Gothenburg, Sweden) at a final concentration of 2% to enable transfer of the sample. For analysis of platelet counts blood was collected in microvettes with EDTA (Sarstedt, Nümbrecht, Germany) and fixed in 0.5% PFA. Platelet counts were assayed using a Sysmex Kx-21N system according to the manufacturer’s instructions (Sysmex America, Mundelein, IL). Platelet counts and BUN are presented in S1 Fig. Kidneys were fixed overnight in 2.5% glutaraldehyde in 0.15 M sodium-cacodylate buffer (pH 7.9).

### Intraperitoneal injection of Stx2

Purified Stx2 (obtained from C Thorpe, Phoenix Lab, Tufts Medical Center, Boston, MA) was diluted in PBS and injected intraperitoneally at a dose 285 pg/g weight as previously described [8]. In this model symptoms usually develop on day 3–5 after injection. At day 1, 2, 3 or 4 blood was collected from vena saphena and by heart puncture after isoflurane anesthesia and the animals were then sacrificed by cervical dislocation. Blood samples for analysis of microvesicles were treated as described above.

### Isolation, detection and identification of murine microvesicles

Murine microvesicles was purified from whole blood fixed in 2% PFA by centrifugation for 15 min at 1500g at 20°C to remove the pellet of blood cells. The platelet-poor-plasma supernatant was collected and further centrifuged at 13000g for 3 min to obtain platelet-free-plasma in the supernatant. The sample was diluted in sterile-filtered PBS and further centrifuged for 45 min at 24000g at 15°C to obtain a precipitate containing a microvesicle-enriched suspension.

Identification of blood cell-derived microvesicles in murine plasma was carried out as per S2 Table (in supporting information). Using flow cytometry microvesicles were identified by incubation of the enriched suspension with a mixture of rat anti-mouse CD41:APC (1:40, detects platelets), rat anti-mouse CD45R/B220:PerCP-Cy5.5 (1:300, detects monocytes, B-cells, NK-cells and T-cells) and rat anti-mouse Ly-6G:PE (1:300, detects granulocytes and monocytes) or isotype controls IgG_1_:APC, IgG_2a_:PE or IgG_2a_:PerCP-Cy5.5 (all from BD Biosciences, San Diego, CA) for 20 min at rt. Events staining positively for both CD45R/B220 and Ly-6G were considered to represent microvesicles released from monocytes while events staining for Ly-6G alone were considered to represent microvesicles released from neutrophils.

Stx2 was detected by incubation with polyclonal rabbit anti-Shiga toxin (Stx)2 B-subunit (1:200, BEI Resources, Manassas, VA, diluted in 0.1% saponin (Sigma-Aldrich) to enable intravesicular staining of Stx2). Rabbit IgG (eBioscience, San Diego, CA) was used as the negative control and swine anti-rabbit:FITC F(ab´)_2_ (1:300, Dako, Glostrup, Denmark) as the secondary antibody.

### Transmission electron microscopy of human and murine kidneys

Renal tissue sections from the HUS patient were embedded and subject to antigen retrieval with metaperiodate[63]. Grids were blocked with 5% (v/v) goat serum diluted in 0.2% bovine serum albumin (pH 7.6, Aurion, Wageningen, Netherlands) for 15 min followed by incubation with polyclonal rabbit anti-Stx2 B-subunit (1:80) and mouse anti-human CD42b (1:80, to detect platelets) or mouse anti-human CD45 (1:100, to detect leukocytes, both from BioLegend, San Diego, CA) overnight at 4°C. Samples were then incubated with gold-conjugated goat anti-rabbit IgG:5nm (1:10) or goat anti-mouse IgG:10nm (1:20, both from BBI, Cardiff, UK) for 1h at rt followed by fixation in 2% glutaraldehyde and post-stained with uranyl acetate and lead citrate. Rabbit IgG or mouse IgG (BioLegend) were used as negative controls.

Similarly, renal tissues from mice were fixed and sectioned as described above and incubated with rabbit anti-Stx2 (1:80), rat anti-mouse CD41 (1:100, detects platelets) or rat anti-mouse CD18 (1:100, detects leukocytes, both from eBioscience) and gold-conjugated reagents as described above. Rabbit IgG, rat IgG_1_ or rat IgG_2b_ (all from eBioscience) was used as negative controls.

Sections were examined with a transmission electron microscope (CM100 Twin, Philips, Eindhoven, Holland) operated at a 60 kV accelerating voltage. The images were recorded with a side-mounted Olympus Veleta camera (Olympus, Münster, Germany).

### Stimulation of human whole blood or purified red blood cells with Stx2

Whole blood diluted 1:2 in LPS-free DMEM (Invitrogen, Paisley, UK) containing Gly-Pro-Arg-Asp (GPRP, 10 μM, Sigma-Aldrich) was incubated with purified Stx2 (200 ng/mL, a gift from T.G. Obrig, Department of Microbiology and Immunology, University of Maryland, Baltimore or obtained from C. Thorpe as above) or the catalytically inactive Stx2-mutant (mutated in the enzymatic A subunit active site [64] 200 ng/mL, from C. Thorpe) for 1h. The LPS content of the Stx2 preparation (from T.G. Obrig) was assayed by the limulus amebocyte assay (Coatex, Gothenburg Sweden) and found to be less than 50 pg/ml (the detection limit) and the preparations from C Thorpe were assayed by Endochrome 140 (Charles River, L’Arbresle Cedex, France) and found to be 25 ng/mL (300 endotoxin units/mL). For electron microscopy experiments whole blood was first incubated with Stx2 for 1h followed by incubation with a calcium ionophore (A23187, 10 μM, Sigma-Aldrich) for 30 min to increase the total numbers of microvesicles.

Red blood cells (RBCs) were isolated immediately after blood collection by centrifugation (830g, 5 min, 20°C), and washed three times in PBS. The RBCs (4.5x10^8^/mL) were diluted 1:2 in RBC-donor specific citrated plasma and LPS-free RPMI1640 (Invitrogen) and incubated with Stx2 for 40 min at 37°C under gentle shaking. All samples were analyzed for the release of microvesicles containing Stx2 by flow cytometry, as described below.

### Isolation of human microvesicles

Microvesicles were isolated from patient or control platelet-free-plasma or from in vitro stimulation experiments (whole blood and RBC stimulation) as previously described [27] with the following modifications. Aliquots of platelet-free-plasma (200 μL) were thawed in a 37°C water bath for 5 min and centrifuged at 20800g for 10 min at 7°C. 150 μL of the supernatant were discarded and the remaining 50 μL were washed twice (for flow cytometry) or five times (for the cytotoxicity assay and electron microscopy) in sterile Hank’s Balanced Salt Solution without Ca^2+^ and Mg^2+^ (HBSS, PAA Laboratories GMBH, Pasching, Austria) and centrifuged as above. For flow cytometry assay microvesicles were fixed in 1% PFA for 30 min. To reduce background all buffers, cell media and PFA were filtered through a 0.2 μm pore-sized filter (Pall Corporation, Ann Arbor, MI).

### Labeling of microvesicles with cell-specific antibodies

Subpopulations of blood cell-derived microvesicles were determined by incubation with mouse anti-human CD42b:APC (1:20), mouse anti-human CD38:PerCP-Cy5.5 (1:100, detects monocytes), mouse anti-human CD66:PE (1:40, neutrophils) or mouse anti-human CD235a:PE (1:800, RBCs) for 30 min in the dark. Mouse IgG_1_:APC, mouse IgG_1_:PerCP-Cy5.5, mouse IgG_1_:PE or mouse IgG_2b_:PE were used as iso-type controls (all from BD Biosciences).

### Detection of Stx2 in microvesicles by flow cytometry

Microvesicles from patients or in vitro experiments were fixed in 1% PFA and incubated with polyclonal rabbit anti-Stx2 B-subunit (1:200, used in whole blood experiments) or monoclonal mouse anti-Stx2 IgG_1_ (11E10, 200 ng/mL, a gift from T.G. Obrig, used in RBC experiments) for 30 min. The choice of anti-Stx used depended on the species origin of the microvesicles. Rabbit IgG (eBioscience) or mouse IgG1k (Dako) were used as negative controls and swine anti-rabbit:FITC F(ab´)_2_ (1:300, Dako) or goat anti-mouse:FITC (1:1000, Dako) as the secondary antibodies. All antibodies were diluted in 0.1% sterile-filtered saponin/PBS for detection of toxin within microvesicles. To detect Stx2 on the surface of the microvesicles certain experiments were carried out without saponin.

### Acquisition and analysis of microvesicles by flow cytometry

Samples were analyzed using a FACSCanto^TM^II flow cytometer with FACSDiva software version 6.0 (BD Immunocytometry Systems, San Jose, CA) or a CyFlow Cube 8 flow cytometer (Partec, Görlitz, Germany) with FCS Express 4 Flow Research Edition software version 4.07.0003 (De Novo Software, Glendale, CA). Microvesicles were defined by size and positive fluorescence using cell-specific antibodies. Both forward- (FSC) and side scatter (SSC) signals were recorded with logarithmic gain and flow rate was set to low. The microvesicle gate was generated using 0.5, 0.9 and 3.0 μm beads (Megamix beads, BioCytex, Marseille, France) to determine upper limits in both FSC (280) and SSC (340) signals and the lower limits were placed above the background level of the machine and/or buffer which was determined by running 0.2 μm-filtered HBSS.

Single-stained controls were used to check fluorescence compensation settings and fluorescence-minus-one (FMO) was used to define events with fluorescence above background levels to set up positive regions. A microvesicle was defined as an event positive for a specific cell marker and ≤1 μm in size. Microvesicles were quantified as previously described [27].

### Cell culture

Conditionally immortalized glomerular cells (CiGEnC) were obtained from Dr. Simon Satchell (Academic Renal Unit, University of Bristol, UK), and cultured as described [65]. CiGEnC were used at passage 26–36. Cells were grown in endothelial growth medium 2—microvascular (EGM2-MV) supplemented with 5% fetal bovine serum and growth factors (all from Lonza, Walkersville, MD) as well as 100 U/mL penicillin and 100 μg/mL streptomycin (PAA Laboratories Gmbh). Cells were grown to 80–90% confluence at 33°C in 5% CO_2_ and then allowed to differentiate for at least five days at 37°C.

### Transfer of Stx2-containing microvesicles to glomerular endothelial cells and imaging

CiGEnC were cultured in T25 culture flasks (TPP AG, Trasadingen, Switzerland) and grown to 90% confluence. The cells were washed with Ca^2+^/Mg^2+^ free HBSS and incubated with Stx2-containing microvesicles isolated from 10 mL whole blood/DMEM. Microvesicles were diluted 1:10 in the cell culture media described above and incubated with the cells for 1, 3, 12 or 24 hours, respectively. Cells were washed with Ca^2+^ /Mg^2+^ free HBSS and detached from the culture flasks using 1x TrypLE^TM^ Select (Life Technology, Carlsbad, CA), washed and fixed in 2.5% glutaraldehyde in 0.15 M sodium-cacodylate buffer (pH 7.9) for 24h. Samples were then dehydrated and prepared for electron microscopy. After overnight polymerization, ultra-thin sections were cut and incubated with polyclonal rabbit anti-Stx2 B-subunit (1:80) and mouse anti-human CD42b (1:80) or mouse anti-human CD45 (1:100). To detect early endosomes or ribosomes samples were incubated with rabbit anti-Rab5 antibody (1:50, Abcam, Cambridge UK) or mouse anti-rRNA Antibody (1:100, Thermo Fisher Scientific Inc., USA), respectively. Samples were then incubated with gold-conjugated goat anti-rabbit IgG:5nm to label Stx or goat anti-mouse IgG:10nm to label platelet or leukocyte microvesicle membranes and goat anti-rabbit IgG:20nm or goat anti-mouse IgG:20nm to label early endosomes or ribosomes, respectively (BBI). Rabbit IgG or mouse IgG (BioLegend) were used as negative controls.

### Cytotoxicity assay

CiGEnC were plated in 96-well plates (Nunc, Roskilde, Denmark) at a density of 1x10^4^ cells/well and incubated as described above. The cells were washed in Ca^2+^/Mg^2+^-free HBSS and pre-incubated with cycloheximide (300 μg/mL, Sigma-Aldrich) in order to enhance protein synthesis-inhibition induced by Shiga toxin, as previously described [66], diluted in EGM2-MV for 3h, washed with HBSS and incubated with microvesicles (2x10^5^/well, diluted in EGM2-MV, the quantity was determined by flow cytometry). Microvesicles were isolated from Stx2- or Stx2 mutant-treated, or untreated whole blood for 36h. All microvesicle samples were washed five times in HBSS. Control samples were treated with Stx2 under two conditions. In the one Stx2 (200 ng/mL, diluted in EGM2-MV) was exposed to washing steps similar to microvesicles before exposure to the cells and in the other cells were exposed to pure Stx2 (200 ng/mL, final concentration diluted in EGM2-MV) without washing steps. Cell viability was determined by crystal violet staining [67] and absorbance measured at 570nm. The effect on cell viability was calculated as the difference in absorbance percentage in the presence or absence of microvesicles. All experiments were performed in triplicate and repeated five times.

### Protein synthesis assay

Protein synthesis was determined by assaying the incorporation of [^35^S]-methionine into newly synthesized proteins. CiGEnC were grown to 80–90% confluence in 24-well plates (Becton Dickinson, Lincoln Park, NJ) as described above. The cells were washed in Ca^2+^ /Mg^2+^ free HBSS and incubated with cycloheximide for 3 h. Subsequently, the cells were washed and incubated with microvesicles (1 x 10^6^/well, diluted in EGM2-MV) isolated from Stx2-stimulated or unstimulated whole blood, for 36 h, after which the cells were pulsed with [^35^S] methionine (50 μCi/mL, PerkinElmer, Waltham, MA), diluted in methionine-free DMEM (Life Technology) for 2 h. After incubation, the cells were washed with Ca^2+^ /Mg^2+^-free HBSS, detached with TrypLe and lysed in a radioimmune precipitation assay (RIPA) buffer (0.15 M NaCl, 30mM HEPES, pH 7.3, 1% Triton-X (v/v), 1% sodium deoxycholate (w/v), and 1% SDS (w/v)) at-20°C for at least one h. Proteins were precipitated with 55% trichloroacetic acid (TCA, Fisher Scientific, Leicestershire, UK), washed with acetone and resuspended in 0.1 M Tric-HCl, pH 8. The proteins were added to liquid scintillation fluid and counted in a liquid scintillation counter. All experiments were done in duplicate and repeated three times (large amounts of blood (>100 ml/analysis) were required for the microvesicle isolation resulting in three experiments and precluding statistical analysis). Inhibition of protein synthesis was defined as a decrease in the ability of the cells to incorporate [^35^S]-methionine divided by total protein.

### Statistics

Differences between patients and controls or differences between stimulated and unstimulated samples were assayed by the non-parametrical Mann-Whitney test. All data were analyzed using GraphPadPrism 6.0 (GraphPad Software, Inc., La Jolla, CA). *P* values of ≤0.05 were considered significant.

### Ethics statement

Samples from patients, pediatric controls and healthy donors were taken with the informed written consent of the subjects or their parents. All children had consent given by a parent/guardian as well as their own consent, if over 15 years of age. All adult participants provided their own written informed consent. The study was performed with the approval of the Ethics committee of the Medical Faculty, Lund University (permit #323-2006).

All animal experiments were approved by the Laboratory Animal Ethics Committee of Lund University (permit #M391-12) in accordance to guidelines of the Swedish National Board of Agriculture and the EU directive for the protection of animals used for scientific purposes.

## Supporting Information

S1 Table Characteristics of patients included in this study. (DOCX)

S2 Table Identification of blood cell-derived microvesicles in murine plasma. (DOCX)

S1 Fig Plaletet counts and blood urea nitrogen in infected mice. Platelet counts and blood urea nitrogen (BUN) levels in mice infected with *E. coli* O157:H7 strains 86–24 (Shiga toxin 2-producing) or 87–23 (non Shiga toxin-producing) depicted as days after inoculation.
(PPTX)

S2 Fig Detailed enlargement of microvesicles demonstrated in Fig. 3 panels I-N. The upper panels are areas from Fig. 3 I-N. Scale bar 100 nm. The lower panels are enlargements of the boxed areas within each of the respective upper panels. Samples were co-incubated with rabbit anti-Stx2 (5 nm, smaller gold conjugates) and rat anti-mouse CD41 (10 nm, detects platelet-derived microvesicles) or rat anti-mouse CD45 (10 nm, detects leukocyte-derived microvesicles, larger gold conjugates).
(TIF)

## References

[1] McKee ML, O'Brien AD (1995) Investigation of enterohemorrhagic *Escherichia coli* O157:H7 adherence characteristics and invasion potential reveals a new attachment pattern shared by intestinal *E. coli* . Infect Immun 63: 2070–2074. 7537254 10.1128/iai.63.5.2070-2074.1995 PMC173266

[2] Habib R (1992) Pathology of the Hemolytic Uremic Syndrome In: Kaplan BS, Trompeter R.S., Moake J.L., editor. Hemolytic Uremic Syndrome and Thrombotic thrombocytopenic purpura. New York: Marcel Dekker Inc.

[3] Karpman D, Håkansson A, Perez MT, Isaksson C, Carlemalm E, et al (1998) Apoptosis of renal cortical cells in the hemolytic-uremic syndrome: in vivo and in vitro studies. Infect Immun 66: 636–644. 9453620 10.1128/iai.66.2.636-644.1998 PMC107951

[4] Wadolkowski EA, Sung LM, Burris JA, Samuel JE, O'Brien AD (1990) Acute renal tubular necrosis and death of mice orally infected with *Escherichia coli* strains that produce Shiga-like toxin type II. Infect Immun 58: 3959–3965. 2254023 10.1128/iai.58.12.3959-3965.1990 PMC313762

[5] Karpman D, Connell H, Svensson M, Scheutz F, Alm P, et al (1997) The role of lipopolysaccharide and Shiga-like toxin in a mouse model of *Escherichia coli* O157:H7 infection. J Infect Dis 175: 611–620. 9041333 10.1093/infdis/175.3.611

[6] Chromek M, Arvidsson I, Karpman D (2012) The antimicrobial peptide cathelicidin protects mice from *Escherichia coli* O157:H7-mediated disease. PLoS One 7: e46476 10.1371/journal.pone.0046476 23077510 PMC3471911

[7] Psotka MA, Obata F, Kolling GL, Gross LK, Saleem MA, et al (2009) Shiga toxin 2 targets the murine renal collecting duct epithelium. Infect Immun 77: 959–969. 10.1128/IAI.00679-08 19124603 PMC2643625

[8] Calderon Toledo C, Rogers TJ, Svensson M, Tati R, Fischer H, et al (2008) Shiga toxin-mediated disease in MyD88-deficient mice infected with *Escherichia coli* O157:H7. Am J Pathol 173: 1428–1439. 10.2353/ajpath.2008.071218 18832584 PMC2570133

[9] Békássy ZD, Calderon Toledo C, Leoj G, Kristoffersson A, Leopold SR, et al (2011) Intestinal damage in enterohemorrhagic *Escherichia coli* infection. Pediatr Nephrol 26: 2059–2071. 10.1007/s00467-010-1616-9 20809220

[10] Lindberg AA, Brown JE, Strömberg N, Westling-Ryd M, Schultz JE, et al (1987) Identification of the carbohydrate receptor for Shiga toxin produced by *Shigella dysenteriae* type 1. J Biol Chem 262: 1779–1785. 3543013

[11] Sandvig K, Garred O, Prydz K, Kozlov JV, Hansen SH, et al (1992) Retrograde transport of endocytosed Shiga toxin to the endoplasmic reticulum. Nature 358: 510–512. 1641040 10.1038/358510a0

[12] Endo Y, Tsurugi K, Yutsudo T, Takeda Y, Ogasawara T, et al (1988) Site of action of a Vero toxin (VT2) from *Escherichia coli* O157:H7 and of Shiga toxin on eukaryotic ribosomes. RNA N-glycosidase activity of the toxins. Eur J Biochem 171: 45–50. 3276522 10.1111/j.1432-1033.1988.tb13756.x

[13] Waddell T, Cohen A, Lingwood CA (1990) Induction of verotoxin sensitivity in receptor-deficient cell lines using the receptor glycolipid globotriosylceramide. Proc Natl Acad Sci U S A 87: 7898–7901. 2236008 10.1073/pnas.87.20.7898 PMC54858

[14] Chark D, Nutikka A, Trusevych N, Kuzmina J, Lingwood C (2004) Differential carbohydrate epitope recognition of globotriaosyl ceramide by verotoxins and a monoclonal antibody. Eur J Biochem 271: 405–417. 14717708 10.1046/j.1432-1033.2003.03941.x

[15] Jacewicz MS, Mobassaleh M, Gross SK, Balasubramanian KA, Daniel PF, et al (1994) Pathogenesis of Shigella diarrhea: XVII. A mammalian cell membrane glycolipid, Gb3, is required but not sufficient to confer sensitivity to Shiga toxin. J Infect Dis 169: 538–546. 8158025 10.1093/infdis/169.3.538

[16] Khan F, Proulx F, Lingwood CA (2009) Detergent-resistant globotriaosyl ceramide may define verotoxin/glomeruli-restricted hemolytic uremic syndrome pathology. Kidney Int 75: 1209–1216. 10.1038/ki.2009.7 19212418

[17] Schüller S, Frankel G, Phillips AD (2004) Interaction of Shiga toxin from *Escherichia coli* with human intestinal epithelial cell lines and explants: Stx2 induces epithelial damage in organ culture. Cell Microbiol 6: 289–301. 14764112 10.1046/j.1462-5822.2004.00370.x

[18] Petruzziello-Pellegrini TN, Yuen DA, Page AV, Patel S, Soltyk AM, et al (2012) The CXCR4/CXCR7/SDF-1 pathway contributes to the pathogenesis of Shiga toxin-associated hemolytic uremic syndrome in humans and mice. J Clin Invest 122: 759–776. 10.1172/JCI57313 22232208 PMC3266777

[19] Morigi M, Galbusera M, Gastoldi S, Locatelli M, Buelli S, et al (2011) Alternative pathway activation of complement by Shiga toxin promotes exuberant C3a formation that triggers microvascular thrombosis. J Immunol 187: 172–180. 10.4049/jimmunol.1100491 21642543

[20] Keepers TR, Psotka MA, Gross LK, Obrig TG (2006) A murine model of HUS: Shiga toxin with lipopolysaccharide mimics the renal damage and physiologic response of human disease. J Am Soc Nephrol 17: 3404–3414. 17082244 10.1681/ASN.2006050419

[21] Brigotti M, Tazzari PL, Ravanelli E, Carnicelli D, Rocchi L, et al (2011) Clinical relevance of shiga toxin concentrations in the blood of patients with hemolytic uremic syndrome. Pediatr Infect Dis J 30: 486–490. 10.1097/INF.0b013e3182074d22 21164386

[22] Ståhl AL, Sartz L, Nelsson A, Békássy ZD, Karpman D (2009) Shiga toxin and lipopolysaccharide induce platelet-leukocyte aggregates and tissue factor release, a thrombotic mechanism in hemolytic uremic syndrome. PLoS One 4: e6990 10.1371/journal.pone.0006990 19750223 PMC2735777

[23] Brigotti M, Caprioli A, Tozzi AE, Tazzari PL, Ricci F, et al (2006) Shiga toxins present in the gut and in the polymorphonuclear leukocytes circulating in the blood of children with hemolytic-uremic syndrome. J Clin Microbiol 44: 313–317. 16455876 10.1128/JCM.44.2.313-317.2006 PMC1392687

[24] Brigotti M, Tazzari PL, Ravanelli E, Carnicelli D, Barbieri S, et al (2010) Endothelial damage induced by Shiga toxins delivered by neutrophils during transmigration. J Leukoc Biol 88: 201–210. 10.1189/jlb.0709475 20371598

[25] te Loo DM, Monnens LA, van Der Velden TJ, Vermeer MA, Preyers F, et al (2000) Binding and transfer of verocytotoxin by polymorphonuclear leukocytes in hemolytic uremic syndrome. Blood 95: 3396–3402. 10828021

[26] Karpman D, Papadopoulou D, Nilsson K, Sjögren AC, Mikaelsson C, et al (2001) Platelet activation by Shiga toxin and circulatory factors as a pathogenetic mechanism in the hemolytic uremic syndrome. Blood 97: 3100–3108. 11342436 10.1182/blood.v97.10.3100

[27] Ståhl AL, Sartz L, Karpman D (2011) Complement activation on platelet-leukocyte complexes and microparticles in enterohemorrhagic *Escherichia coli*-induced hemolytic uremic syndrome. Blood 117: 5503–5513. 10.1182/blood-2010-09-309161 21447825

[28] Hugel B, Martinez MC, Kunzelmann C, Freyssinet JM (2005) Membrane microparticles: two sides of the coin. Physiology (Bethesda) 20: 22–27. 15653836 10.1152/physiol.00029.2004

[29] Simak J, Gelderman MP (2006) Cell membrane microparticles in blood and blood products: potentially pathogenic agents and diagnostic markers. Transfus Med Rev 20: 1–26. 16373184 10.1016/j.tmrv.2005.08.001

[30] Mack M, Kleinschmidt A, Bruhl H, Klier C, Nelson PJ, et al (2000) Transfer of the chemokine receptor CCR5 between cells by membrane-derived microparticles: a mechanism for cellular human immunodeficiency virus 1 infection. Nat Med 6: 769–775. 10888925 10.1038/77498

[31] Rozmyslowicz T, Majka M, Kijowski J, Murphy SL, Conover DO, et al (2003) Platelet- and megakaryocyte-derived microparticles transfer CXCR4 receptor to CXCR4-null cells and make them susceptible to infection by X4-HIV. AIDS 17: 33–42. 12478067 10.1097/00002030-200301030-00006

[32] Mause SF, von Hundelshausen P, Zernecke A, Koenen RR, Weber C (2005) Platelet microparticles: a transcellular delivery system for RANTES promoting monocyte recruitment on endothelium. Arterioscler Thromb Vasc Biol 25: 1512–1518. 15890969 10.1161/01.ATV.0000170133.43608.37

[33] Risitano A, Beaulieu LM, Vitseva O, Freedman JE (2012) Platelets and platelet-like particles mediate intercellular RNA transfer. Blood 119: 6288–6295. 10.1182/blood-2011-12-396440 22596260 PMC3383198

[34] Diehl P, Fricke A, Sander L, Stamm J, Bassler N, et al (2012) Microparticles: major transport vehicles for distinct microRNAs in circulation. Cardiovasc Res 93: 633–644. 10.1093/cvr/cvs007 22258631 PMC3291092

[35] Ge S, Hertel B, Emden SH, Beneke J, Menne J, et al (2012) Microparticle generation and leucocyte death in Shiga toxin-mediated HUS. Nephrol Dial Transplant 27: 2768–2775. 10.1093/ndt/gfr748 22234918

[36] Meckes DG Jr., Raab-Traub N (2011) Microvesicles and viral infection. J Virol 85: 12844–12854. 10.1128/JVI.05853-11 21976651 PMC3233125

[37] Bauwens A, Betz J, Meisen I, Kemper B, Karch H, et al (2013) Facing glycosphingolipid-Shiga toxin interaction: dire straits for endothelial cells of the human vasculature. Cell Mol Life Sci 70: 425–457. 10.1007/s00018-012-1060-z 22766973 PMC11113656

[38] Okuda T, Tokuda N, Numata S, Ito M, Ohta M, et al (2006) Targeted disruption of Gb3/CD77 synthase gene resulted in the complete deletion of globo-series glycosphingolipids and loss of sensitivity to verotoxins. J Biol Chem 281: 10230–10235. 16476743 10.1074/jbc.M600057200

[39] Schüller S (2011) Shiga toxin interaction with human intestinal epithelium. Toxins (Basel) 3: 626–639. 10.3390/toxins3060626 22069729 PMC3202847

[40] Malyukova I, Murray KF, Zhu C, Boedeker E, Kane A, et al (2009) Macropinocytosis in Shiga toxin 1 uptake by human intestinal epithelial cells and transcellular transcytosis. Am J Physiol Gastrointest Liver Physiol 296: G78–92. 10.1152/ajpgi.90347.2008 18974311 PMC2636932

[41] Griener TP, Mulvey GL, Marcato P, Armstrong GD (2007) Differential binding of Shiga toxin 2 to human and murine neutrophils. J Med Microbiol 56: 1423–1430. 17965340 10.1099/jmm.0.47282-0

[42] Arfilli V, Carnicelli D, Rocchi L, Ricci F, Pagliaro P, et al (2010) Shiga toxin 1 and ricin A chain bind to human polymorphonuclear leucocytes through a common receptor. Biochem J 432: 173–180. 10.1042/BJ20100455 20809900

[43] Cooling LL, Walker KE, Gille T, Koerner TA (1998) Shiga toxin binds human platelets via globotriaosylceramide (Pk antigen) and a novel platelet glycosphingolipid. Infect Immun 66: 4355–4366. 9712788 10.1128/iai.66.9.4355-4366.1998 PMC108526

[44] van Setten PA, Monnens LA, Verstraten RG, van den Heuvel LP, van Hinsbergh VW (1996) Effects of verocytotoxin-1 on nonadherent human monocytes: binding characteristics, protein synthesis, and induction of cytokine release. Blood 88: 174–183. 8704172

[45] Bitzan M, Richardson S, Huang C, Boyd B, Petric M, et al (1994) Evidence that verotoxins (Shiga-like toxins) from *Escherichia coli* bind to P blood group antigens of human erythrocytes in vitro. Infect Immun 62: 3337–3347. 8039905 10.1128/iai.62.8.3337-3347.1994 PMC302964

[46] Fontaine A, Arondel J, Sansonetti PJ (1988) Role of Shiga toxin in the pathogenesis of bacillary dysentery, studied by using a Tox- mutant of *Shigella dysenteriae* 1. Infect Immun 56: 3099–3109. 3053452 10.1128/iai.56.12.3099-3109.1988 PMC259708

[47] Ståhl AL, Svensson M, Mörgelin M, Svanborg C, Tarr PI, et al (2006) Lipopolysaccharide from enterohemorrhagic *Escherichia coli* binds to platelets through TLR4 and CD62 and is detected on circulating platelets in patients with hemolytic uremic syndrome. Blood 108: 167–176. 16514062 10.1182/blood-2005-08-3219 PMC1895830

[48] te Loo DM, Heuvelink AE, de Boer E, Nauta J, van der Walle J, et al (2001) Vero cytotoxin binding to polymorphonuclear leukocytes among households with children with hemolytic uremic syndrome. J Infect Dis 184: 446–450. 11471102 10.1086/322782

[49] te Loo DM, van Hinsbergh VW, van den Heuvel LP, Monnens LA (2001) Detection of verocytotoxin bound to circulating polymorphonuclear leukocytes of patients with hemolytic uremic syndrome. J Am Soc Nephrol 12: 800–806. 11274241 10.1681/ASN.V124800

[50] Brigotti M, Carnicelli D, Arfilli V, Tamassia N, Borsetti F, et al (2013) Identification of TLR4 as the receptor that recognizes Shiga toxins in human neutrophils. J Immunol 191: 4748–4758. 10.4049/jimmunol.1300122 24068665

[51] Falguieres T, Mallard F, Baron C, Hanau D, Lingwood C, et al (2001) Targeting of Shiga toxin B-subunit to retrograde transport route in association with detergent-resistant membranes. Mol Biol Cell 12: 2453–2468. 11514628 10.1091/mbc.12.8.2453 PMC58606

[52] Morel O, Jesel L, Freyssinet JM, Toti F (2011) Cellular mechanisms underlying the formation of circulating microparticles. Arterioscler Thromb Vasc Biol 31: 15–26. 10.1161/ATVBAHA.109.200956 21160064

[53] Karmali MA, Petric M, Lim C, Fleming PC, Arbus GS, et al (1985) The association between idiopathic hemolytic uremic syndrome and infection by verotoxin-producing *Escherichia coli* . J Infect Dis 151: 775–782. 3886804 10.1093/infdis/151.5.775

[54] Caprioli A, Luzzi I, Rosmini F, Pasquini P, Cirrincione R, et al (1992) Hemolytic-uremic syndrome and Vero cytotoxin-producing *Escherichia coli* infection in Italy. The HUS Italian Study Group. J Infect Dis 166: 154–158. 1607689 10.1093/infdis/166.1.154

[55] Porubsky S, Federico G, Muthing J, Jennemann R, Gretz N, et al (2014) Direct acute tubular damage contributes to Shiga toxin-mediated kidney failure. J Pathol 234: 120–133. 10.1002/path.4388 24909663 PMC4282478

[56] Owens AP 3rd, Mackman N (2011) Microparticles in hemostasis and thrombosis. Circ Res 108: 1284–1297. 10.1161/CIRCRESAHA.110.233056 21566224 PMC3144708

[57] Müller I, Klocke A, Alex M, Kotzsch M, Luther T, et al (2003) Intravascular tissue factor initiates coagulation via circulating microvesicles and platelets. FASEB J 17: 476–478. 12514112 10.1096/fj.02-0574fje

[58] Galli M, Grassi A, Barbui T (1996) Platelet-derived microvesicles in thrombotic thrombocytopenic purpura and hemolytic uremic syndrome. Thromb Haemost 75: 427–431. 8701402

[59] Karpman D, Manea M, Vaziri-Sani F, Ståhl AL, Kristoffersson AC (2006) Platelet activation in hemolytic uremic syndrome. Semin Thromb Hemost 32: 128–145. 16575688 10.1055/s-2006-939769

[60] Calderon Toledo C, Arvidsson I, Karpman D (2011) Cross-reactive protection against enterohemorrhagic *Escherichia coli* infection by enteropathogenic *E. coli* in a mouse model. Infect Immun 79: 2224–2233. 10.1128/IAI.01024-10 21402761 PMC3125830

[61] Gagnon M, Kheadr EE, Dabour N, Richard D, Fliss I (2006) Effect of *Bifidobacterium thermacidophilum* probiotic feeding on enterohemorrhagic *Escherichia coli* O157:H7 infection in BALB/c mice. Int J Food Microbiol 111: 26–33. 16822570 10.1016/j.ijfoodmicro.2006.04.041

[62] Palermo M, Alves-Rosa F, Rubel C, Fernandez GC, Fernandez-Alonso G, et al (2000) Pretreatment of mice with lipopolysaccharide (LPS) or IL-1beta exerts dose-dependent opposite effects on Shiga toxin-2 lethality. Clin Exp Immunol 119: 77–83. 10606967 10.1046/j.1365-2249.2000.01103.x PMC1905548

[63] Stirling JW, Graff PS (1995) Antigen unmasking for immunoelectron microscopy: labeling is improved by treating with sodium ethoxide or sodium metaperiodate, then heating on retrieval medium. J Histochem Cytochem 43: 115–123. 7529784 10.1177/43.2.7529784

[64] Stone SM, Thorpe CM, Ahluwalia A, Rogers AB, Obata F, et al (2012) Shiga toxin 2-induced intestinal pathology in infant rabbits is A-subunit dependent and responsive to the tyrosine kinase and potential ZAK inhibitor imatinib. Front Cell Infect Microbiol 135: 1–11. 10.3389/fcimb.2012.00135 PMC3492723 23162799

[65] Satchell SC, Tasman CH, Singh A, Ni L, Geelen J, et al (2006) Conditionally immortalized human glomerular endothelial cells expressing fenestrations in response to VEGF. Kidney Int 69: 1633–1640. 16557232 10.1038/sj.ki.5000277

[66] Petric M, Karmali MA, Arbus GS, Roscoe M, Louie S, et al (1987) Effects of cycloheximide and puromycin on cytotoxic activity of *Escherichia coli* verocytotoxin (Shiga-like toxin). J Clin Microbiol 25: 1265–1268. 3301891 10.1128/jcm.25.7.1265-1268.1987 PMC269190

[67] Gentry MK, Dalrymple JM (1980) Quantitative microtiter cytotoxicity assay for Shigella toxin. J Clin Microbiol 12: 361–366. 7012172 10.1128/jcm.12.3.361-366.1980 PMC273591

