# Supplementary material for: A Novel Mechanism of Bacterial Toxin Transfer within Host Blood Cell-Derived Microvesicles
Source: PLoS Pathog. 2015 Feb 26;11(2):e1004619. doi: 10.1371/journal.ppat.1004619 (PMC4342247; doi:10.1371/journal.ppat.1004619)
Supplement: S1 Table — (DOCX) [file ppat.1004619.s001.docx]

**Table S1.** Characteristics of patients included in this study

| **Patient** | **Sex** | **Age at diagnosis** | **HUS**  **(+/-)** | **Bloody diarrhea**  **(+/-)** | **Dialysis** | **Extra renal symptoms** | ***E. coli* serotype** |
| --- | --- | --- | --- | --- | --- | --- | --- |
| 1 | F | 1 | + | + | + | - | Stx2+ eae  Non-O157 |
| 2 | M | 1 | + | **+** | **-** | **-** | O26 |
| 3 | F | 1 | + | **+** | **+** | **-** | O157 |
| 4 | F | 7 | + | **+** | **+** | **-** | Non-O157 |
| 5 | M | 1 | + | **+** | **+** | Seizures | O153 |
| 6 | M | 10 | + | **+** | **+** | Coma, hemiplegia, seizures | O157 |
| 7 | M | 5 | + | **+** | **+** | **-** | O157 |
| 8 | M | 3 | + | **+** | **-** |  | O157 |
| 9 | M | 7 | + | **-** | **+** |  | O157 |
| 10 | M | 10 | + | **-** | **+** |  | O157 |
| 11 | M | 1 | + | **+** | **-** |  | O26 |
| 12 | M | 2 | + | **+** | **-** |  | O145 |
| 13 | F | 29 | + | - | **-** | **-** | O104 |
| 14 | M | 13 | + | No diarrhea vomiting | **+** |  | O157 |
| 15 | F | 2 | - | **+** | **-** | **-** | O157 |
| 16 | F | 6 | - | **-** | **-** | **-** | Non-O157 |
| 17 | F | 6 | - | **+** | **-** | **-** | O157 Stx1 and Stx2 |
| 18 | F | 9 | - | **+** | **-** | **-** | O157 |
| 19 | F | 31 | - | **+** | **-** | **-** | O157 |
