# Supplementary material for: A Novel Mechanism of Bacterial Toxin Transfer within Host Blood Cell-Derived Microvesicles
Source: PLoS Pathog. 2015 Feb 26;11(2):e1004619. doi: 10.1371/journal.ppat.1004619 (PMC4342247; doi:10.1371/journal.ppat.1004619)
Supplement: S2 Table — (DOCX) [file ppat.1004619.s002.docx]

**Table S2:** Identification of blood cell-derived microvesicles in murine plasma

|  | **CD41** | **Ly-6G** | **CD45R/B220** |
| --- | --- | --- | --- |
| **Platelets** | **+** | **-** | **-** |
| **Granulocytes** | **-** | **+** | **-** |
| **B-cells** | **-** | **-** | **+** |
| **Monocytes** | **-** | **+** | **+** |
| **T-cells** | **-** | **-** | **+** |
| **NK cells** | **-** | **-** | **+** |
