# Supplementary figures and images for: A Novel Mechanism of Bacterial Toxin Transfer within Host Blood Cell-Derived Microvesicles
Source: PLoS Pathog. 2015 Feb 26;11(2):e1004619. doi: 10.1371/journal.ppat.1004619 (PMC4342247; doi:10.1371/journal.ppat.1004619)

## Slide 1
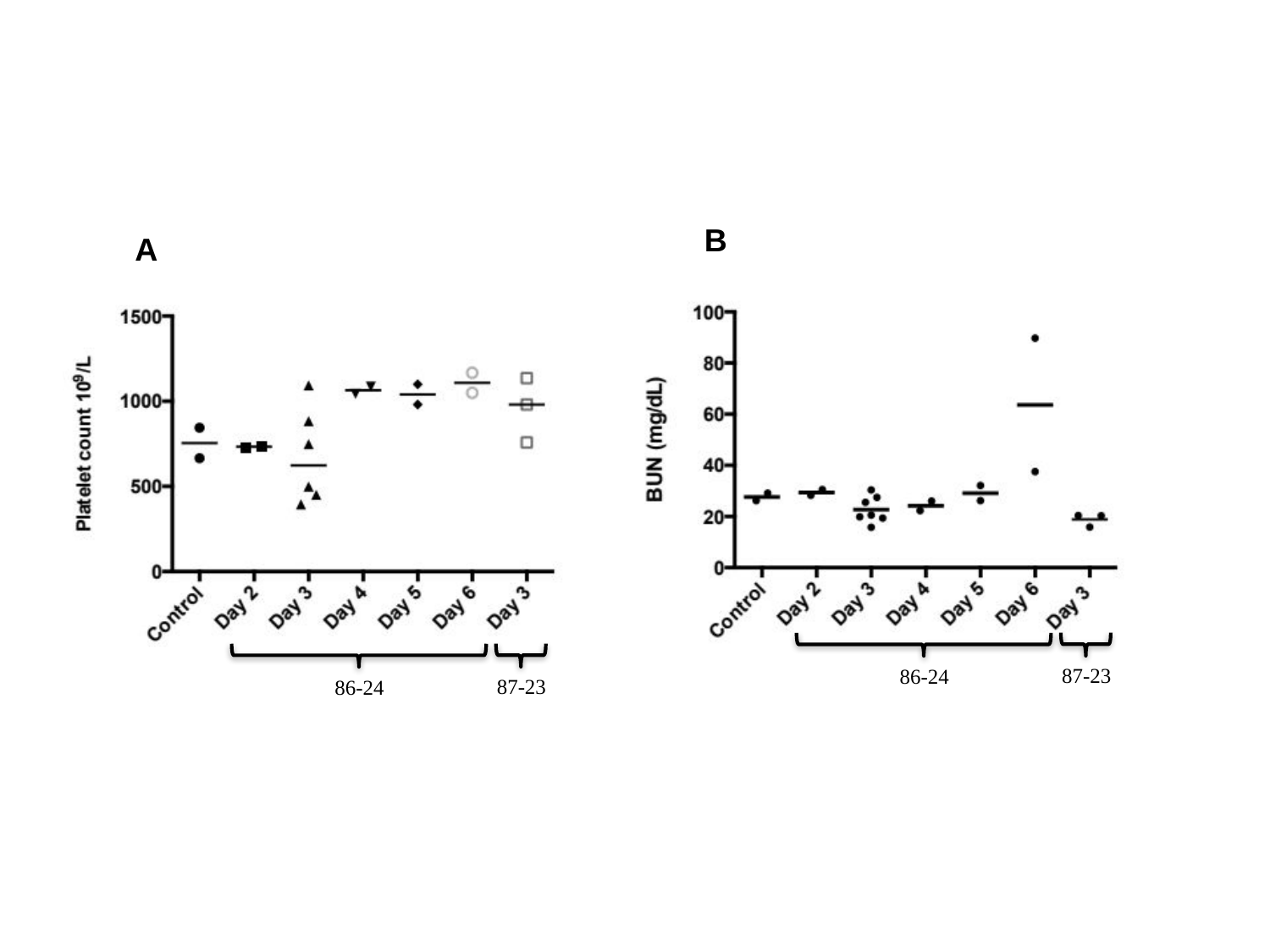

B
A
87-23
86-24
87-23
86-24

Supplement: S1 Fig — Platelet counts and blood urea nitrogen (BUN) levels in mice infected with E. coli O157:H7 strains 86–24 (Shiga toxin 2-producing) or 87–23 (non Shiga toxin-producing) depicted as days after inoculation. (PPTX) [file ppat.1004619.s003.pptx]

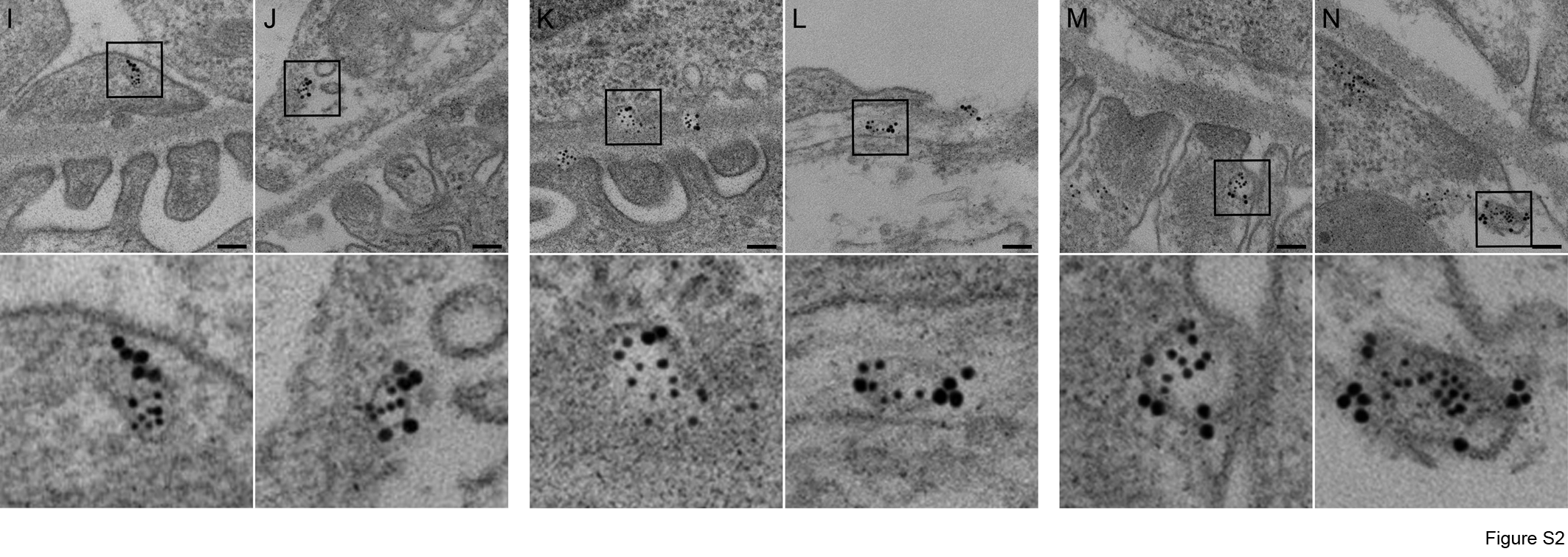

Supplement: S2 Fig — The upper panels are areas from Fig. 3 I-N. Scale bar 100 nm. The lower panels are enlargements of the boxed areas within each of the respective upper panels. Samples were co-incubated with rabbit anti-Stx2 (5 nm, smaller gold conjugates) and rat anti-mouse CD41 (10 nm, detects platelet-derived microvesicles) or rat anti-mouse CD45 (10 nm, detects leukocyte-derived microvesicles, larger gold conjugates). (TIF) [file ppat.1004619.s004.tif]
